# Verification and validation in canine cadaver of an MPI-integrable RF head coil for magnetic particle hyperthermia

**DOI:** 10.1080/02656736.2026.2672017

**Published:** 2026-06-10

**Authors:** Hayden Carlton, Eli Mattingly, Abdul Rahman Mohtasebzadeh, Yash Sharad Lad, Shreeniket Pawar, Anilchandra Attaluri, Dara Kraitchman, Robert Ivkov, Patrick Goodwill

**Affiliations:** a Department of Radiation Oncology and Molecular Radiation Sciences, Johns Hopkins School of Medicine, Baltimore, MD, USA; b Magnetic Insight, Alameda, CA, USA; c Department of Mechanical Engineering, School of Science, Engineering, and Technology, The Pennsylvania State University—Harrisburg, Middletown, PA, USA; d Department of Radiology and Radiological Science, Johns Hopkins School of Medicine, Baltimore, MD, USA; e Department of Molecular and Comparative Pathobiology, Johns Hopkins School of Medicine, Baltimore, MD, USA; f Department of Oncology, Sydney Kimmel Comprehensive Cancer Center, School of Medicine, Johns Hopkins University, Baltimore, MD, USA; g Department of Mechanical Engineering, Whiting School of Engineering, Johns Hopkins University, Baltimore, MD, USA; h Department of Materials Science and Engineering, Whiting School of Engineering, Johns Hopkins University, Baltimore, MD, USA

**Keywords:** Magnetic particle hyperthermia, design verification and validation, magnetic particle imaging, RF coil design, image guided

## Abstract

**Purpose:**

Magnetic particle hyperthermia (MPH), the mild heating of solid tumors with magnetic nanoparticles, is an emerging treatment modality used as an adjuvant to therapies such as ionizing radiation. MPH can be paired with magnetic particle imaging (MPI) hardware to improve thermal treatment planning, spatially confined heating, and thermal dose delivery. In this work, we describe the design, verification, and validation of a radiofrequency (RF) head coil designed to integrate into a clinical MPI scanner.

**Methods:**

The RF coil was designed to accommodate an adult human head, provide a homogeneous (±10%) field along a 10 cm axial centerline, generate a peak magnetic flux density of 10 mT at 100 kHz, and be MPI compliant. Verification of the design consisted of a combination of magnetic field and nanoparticle heating measurements. To validate an MPI/MPH imaging and heating workflow, a canine cadaver was surgically prepared by implanting magnetic nanoparticles in the brain, followed by anatomical imaging, MPI, and heating with the head coil. To explore if the measured performance can be generalized to human treatment, we conducted a virtual MPH treatment of a human glioma.

**Results:**

Coil performance verification demonstrated RF fields as high as *B*_*peak*_ =14 mT at 100.4 kHz, while achieving hyperthermic temperatures in tumor phantoms. The MPI scan of the canine cadaver head revealed differences in signal intensity between particle injection sites, which correlated with temperature increases measured during heating. Computational simulation of a human head showcased the feasibility of this system to treat gliomas.

**Conclusions:**

We successfully constructed, verified, and validated an MPI-compatible RF head coil for combined MPI/MPH therapy.

## 1. Introduction

Hyperthermia is a thermal cancer therapy in which solid tumors are heated to mild temperatures (40–44 C) for a prescribed duration. Early demonstrations of hyperthermia occurred almost a century ago [1,2]. Treatments depend on both time and temperature, with the primary metric for determining the thermal isoeffective dose being cumulative equivalent minutes at 43 C (CEM43) [3–5]. Hyperthermia is typically used as an adjuvant to other therapies such as ionizing radiation [6]. When combined with radiation, hyperthermia increases tumor oxygenation and inhibits DNA damage repair, enhancing efficacy over radiation alone [7].

Magnetic particle hyperthermia (MPH) has received attention since the mid-20^*th*^ century as a targeted hyperthermia modality [8]. In MPH, magnetic nanoparticles (MNPs) are loaded into a tumor through intratumoral or intravenous administration, and heated using an alternating magnetic field (AMF) generated by an external radiofrequency (RF) coil [9]. One MPH application approved in 2010 by the European Medicines Agency was the treatment of recurrent glioblastoma multiforme (GBM) in combination with radiotherapy [10–13]. Assuming that all MNPs are contained within the tumor volume, MPH enables preferential targeting of tumor cells while minimally damaging nearby healthy tissue. Our group has tested several approaches to achieving a consistent thermal dose within the tumor volume while minimizing off-target heating, including temperature feedback control [14,15] and power cycling [16].

While MPH offers unique capabilities for thermal cancer therapy, major challenges remain before it can be widely adopted [17]. Perhaps the most critical challenge is the lack of knowledge relating to the intratumor MNP distribution during MPH therapy. Upon injection, MNPs gradually redistribute, resulting in a heterogeneous delivery within the tumor and nearby healthy tissue. Without knowledge of the MNP distribution, temperatures will be unpredictable, which could lead to inconsistent treatment outcomes.

Our vision to overcome many of the challenges associated with MPH relies on the integration of magnetic particle imaging (MPI), an emerging tracer imaging modality that detects only MNPs and does not detect tissue [18,19]. A combined MPI/MPH system is unique because it utilizes MNPs as both therapeutic and diagnostic agents [20,21]. Thus, MPI provides a mechanism for MPH treatment planning and guidance, where the imaging data can be used to generate predictive computational thermal models [22]. In addition, MPI hardware enables spatially confined heating, that is, heating a pre-selected area, while sparing the surrounding tissue [23–26].

Theranostic MPI/MPH has not yet been demonstrated at a clinical scale, although multiple groups, including ours [27], have developed human-sized MPI systems [28–31]. In order to integrate MPI with MPH, it is necessary to develop an RF coil system compatible with MPI. The RF coil is the central component that drives MPH therapy; it generates the AMF that causes MNPs to dissipate heat via hysteresis losses [32]. While small pre-clinical RF coils (3–10 cm) are ubiquitous, they are primarily used for heating murine animal models and calorimetry studies. Clinical translation requires larger coils (>20 cm in diameter) to accommodate human anatomy. The design requirements for MPI system compatibility differ from simply producing a strong AMF, as the high sensitivity of MPI to magnetic materials precludes the use of flux concentrators such as the ferrite yoke used in the clinically approved MFH-300F system (MagForce AG, Berlin, Germany) [12,33]. Large-scale magnetic induction systems have also been built by AMF Life Systems for pre-clinical work [34–38], but these are also incompatible with MPI. Herein, we describe the design of an efficient, MPI-compatible, RF head coil for MPH therapy, which builds upon our prior preclinical efforts.

The goal of this work was to develop an MPI-compatible RF subsystem, demonstrate an integrated MPI/MPH workflow in a canine cadaver model, and extend these findings to human-relevant scenarios via simulation. The RF system was designed to fit within a clinical MPI scanner, but full integration was beyond the study scope. The results presented here establish a technical path that, with continued development, would enable MPI-guided thermal dose delivery and spatially confined heating.

## 2. Materials and methods

**Figure 1.**
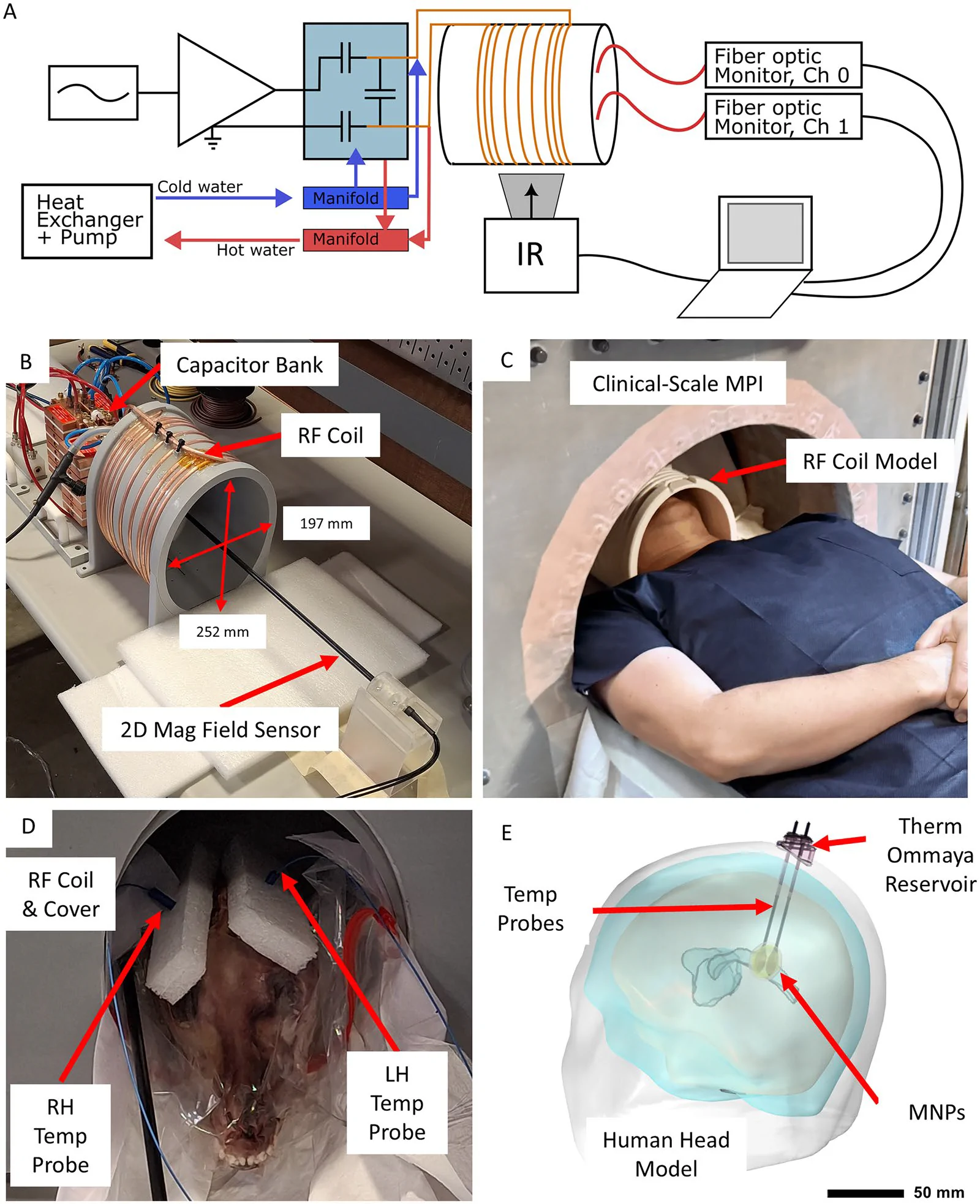
We verified and validated an adult brain RF coil designed to integrate with a clinical MPI scanner. A. Diagram of the MPH system, coil, and experimental setup used in the phantom and cadaver experiments; B. Photo of the head coil, capacitor bank, and AMF probe during field measurements. The coil has major/minor diameters of 252 mm and 197 mm, respectively; C. The coil is shown fitting an adult male positioned within a clinical MPI scanner; D. Picture of the canine cadaver MPH validation experimental setup, including the placement of fiber optic temperature probes in the left (LH) and right (RH) hemispheres; E. A rendered version of the human head computational model used to extend our findings to human-relevant scenarios through simulation.

To achieve the goals of the study, we designed and constructed an adult brain MPH system suitable for integration into a clinical MPI scanner (Figure 1(A,C)) and verified its performance through AMF characterization (Figure 1(B)) and MNP heating experiments in phantoms. We then validated the MPI/MPH workflow by loading a canine cadaver head with MNPs, imaging with computed tomography (CT) and MPI, and performing MNP heating (Figure 1(D)). Last, we performed computational modeling to extend these findings to human-relevant scenarios through simulation (Figure 1(E)). The design, verification, and validation efforts are detailed below.

### 2.1. MPH system design requirements

The instrumentation was designed as an integrated system and guided by four requirements: 1. Support MPH treatment of the adult brain; 2. Provide magnetic field homogeneity of ±10% along a 10 cm axial centerline, positionable over the brain; 3. Require no more than 4.0 kW to generate a peak magnetic flux density (*B*_*peak*_) of 10 mT at 100 kHz; 4. Be suitable for future integration into our clinical MPI scanner [27,39].

### 2.2. MPH hardware and experimental setup

Our system design and MPH experimental setup (Figure 1(A)) was comprised of the following components, presented in order of when they are seen in the signal path: 1. A signal generator and power amplifier; 2. A water-cooled capacitor bank; 3. An RF coil; 4. An infrared (IR) thermal camera and fiber optic temperature probes.

#### 2.2.1. Signal generator and power amplifier

The signal generator and power amplifier drove the RF coil at a single frequency sinusoid, with no amplitude or phase feedback. A signal generator (SDG 1032X, Siglent, Solon, OH) produced a low-voltage signal that served as the input to the power amplifier, which was capable of delivering 4 kW to the load at frequencies from DC-250kHz (8704, AE Techron, Elkhart, IN). The amplifier was power matched to the load at a relatively low impedance of 4 Ω, which contrasts with our prior hyperthermia designs using RF amplifiers that match to a 50 ohm load [24].

#### 2.2.2. Capacitor bank

The capacitor bank power matched the amplifier to the RF coil and included a series capacitor for matching, and a parallel capacitor that resonated with the RF coil (Figure 1(B)). The matching capacitor transformed the impedance of the RF coil at the center frequency to 4 Ω. The resonant capacitor and the RF coil formed the resonant tank. The bank was constructed using capacitors typically used for induction heating and resonant power transfer (CSM 150/300, Celem, Jerusalem, Israel), and were conduction cooled.

#### 2.2.3. RF coil

The RF coil generated the AMF that transferred energy to the MNPs and was designed to be integrable with an MPI scanner, produce a 100 kHz homogeneous magnetic field in the adult brain, and dissipate up to 4 kW. For MPI compatibility, the RF coil used only non-magnetic materials such as plastics and copper and did not use ferrite flux concentrators. The conductor path design was performed in the Biot.jl package (available at https://github.com/EliMattingly22/Biot.jl). The cross-section of the coil was taken to be a loosely fitting ellipse around a typical human head. Numerical optimization to balance homogeneity and power usage led to a coil conductor design with 9 turns, where the centers of the turns had a major and minor diameter of 252 mm and 197 mm, respectively, with an overall length of 137.5 mm from center-center of the outermost turns.

Coil cooling requirements were estimated using the Colebrook equation, which determined the friction factor within the system [40]. At 4 kW, the maximum rise in water temperature would be approximately 24 K for 100 kPa of input water pressure (Flow rate = 39 ml/s, Reynolds number = 13356) at the coil input and 11 K with 400 kPa (Flow rate = 86 ml/s, Reynolds number = 29182).

We simulated the inductance and resistance of the coil with the Biot-Savart solver and Finite Element Method Magnetics (FEMM) 4.2 [41], respectively. For the FEMM model, we assumed a circle with the same area as the ellipse (mean turn radius = 111.4 mm), since the resistance is heavily dependent on proximity and skin effects that are not accounted for using the aforementioned Biot.jl repository. The FEMM simulation uses an improvised asymptotic boundary condition [42] with the default adaptive variable meshing and a simulation diameter of 700 mm centered on the coil. The copper conductivity in FEMM was set to 49.7 MS/m to approximate the conductivity of the alloy (Copper 122) used to construct the coil. We used the model to simulate the coil’s theoretical sensitivity (field per amp). We modeled the field homogeneity as the deviation in the field from the isocenter:

(1) $$Error(x,y,z)=\left(\frac{Simulated(x,y,z)}{Simulated(0,0,0)}-1\right)\times 100$$

The coil was constructed from hollow core copper tubing (6.35 mm outer diameter, 0.76 mm wall thickness) wound on a 3D printed former (Figure 1(B)). We have found that using hollow copper tubing balances power efficiency with ease of construction, as the majority of the RF power is dissipated via resistive losses in the conductor, and the heat can be managed using water pumped through the conductor cavity. The conductor diameter and wall thickness were chosen to balance resistive losses and operating temperature. Because of the small skin depth at 100 kHz of 200 μm, reducing pipe wall thickness can result in a more efficient and cooler system as it allows considerably more water coolant and fewer losses from the proximity effect in excess copper. However, if the walls are too thin, resistive losses can increase. In this design, the tubing wall thickness was chosen to be as close to the analytical optimum of ≈ 0.3 mm [43] subject to commercial availability.

Coil impedance was characterized using an impedance analyzer (E4980AL, Keysight Technologies, Santa Rosa, CA) at 100 kHz. Water cooling was performed with a custom water-water heat exchanger (Boyd, Boca Raton, FL) capable of cooling up to 20 kW at 25°C using deionized water at an operating pressure of up to 600 kPa.

#### 2.2.4. Temperature probes

We performed non-contact and direct contact temperature measurements. For non-contact monitoring of sample temperature and coil surface temperature, we used an IR thermal camera (FLIR E4, *ϵ* = 0.95, Teledyne FLIR, Wilsonville, OR) with a reported accuracy of ±2% of the reading. For direct contact monitoring, we used a pair of fiber optic temperature probes (FOTEMP TS5, Micronor Sensors, Ventura, CA), with a manufacturer reported accuracy of ±0.2°C. Probe temperature offsets were calibrated using a two-phase water/ice mixture at sea-level atmospheric pressure to generate 0°C.

### 2.3. AMF amplitude verification

We measured the field produced by the coil at multiple operating points using a calibrated RF magnetic field probe (2D Magnetic Field Sensor, AMF Life Systems, Auburn Hills, MI). The probe was purchased within two years of the experiment and was operated in accordance with manufacturer guidelines regarding signal interpretation and calibration. The sensor was manually placed (Figure 1(B)) at nine locations along the axial centerline of the coil at 2 cm intervals using a standard tape measure (±1 mm), and presented so that 0 cm designated the axial center. An oscilloscope (DH4204, Rigol, Portland, OR) measured both the signal produced by the calibrated magnetic field probe and the current in the RF coil accurate to ±1% (CWT Rogowski current probe, Power Electronic Measurements, Nottingham, UK). Units of mT throughout this work will refer to the peak magnetic flux density (*B*_*peak*_).

### 2.4. Magnetic nanoparticles

The MNPs used in this study (synomag^®^, micromod Partikeltechnologie, GmbH, Rostock, Germany) were chosen to balance thermal efficiency and MPI resolution, as detailed in our prior work [20]; we utilized various aqueous suspensions of synomag^®^ that we had readily available. For the MNP heating verification experiments, we used synomag-COOH 30 nm (LN: 05125103–03) and synomag-D 70 nm MNPs (LN: 29024104–01). For canine head cadaver heating experiments, we used synomag-COOH 50 nm MNPs (LN: 122622103–01). Throughout this text, we will refer to the MNPs in terms of diameter: 30, 50, or 70 nm. Additionally, all MNPs concentrations refer to the mass of iron per unit volume of liquid provided by the manufacturer.

### 2.5. Nanoparticle heating verification

In addition to verifying the field, verification involved heating MNP suspensions and measuring temperature changes with IR thermometry and fiber optic temperature probes. For these experiments, we were primarily interested in the overall temperature change, so we did not incorporate offset calibration of the temperature probes.

#### 2.5.1. Concentration gradient heating phantom

To test that heating was linear with iron quantity, we created a phantom consisting of four concentrations of 30 nm MNPs held in 25.4 mm plastic spherical vessels on a raised platform. The iron concentration of the MNPs within the vessels were: 6.0 mg/mL, 3.0 mg/mL, 0.6 mg/mL, and 0.0 mg/mL, with the highest concentration being the undiluted MNPs. Dilutions were based on the manufacturer-provided iron concentration of 6.0 mg/mL. The vessels were aligned radially at the axial geometric center of the coil. The IR thermal camera was aligned axially with the coil to provide an orthogonal view of the vessels. The MNP-filled vessels were then heated at *B*_*peak*_ = 11 mT at 100.4 kHz for approximately 15 min, followed by a cooldown to room temperature. Thermal camera images were then analyzed to determine the temperatures of each vessel with respect to time.

#### 2.5.2. Tumor heating phantom

We constructed a basic tumor phantom to model the temperature rise during treatment. The phantom consisted of a 25.4 mm plastic spherical vessel containing 70 nm MNPs at 10 mg/mL immersed in a room temperature deionized water bath. The vessel acted as the spherical “tumor”, surrounded by a uniform temperature aqueous medium or ‘tissue.’ A fiber optic probe was used to measure the temperature of the nanoparticle suspension within the spherical vessel, and a second probe was immersed in the water, roughly halfway between the sphere and the container wall. Heating trials were performed at three peak AMF amplitudes (*B*_*peak*_): 2.5, 5.0, and 10.0 mT, all at a frequency of 100.4 kHz. For all tests, the phantom was heated to steady state before cooling back down to room temperature. In addition, we performed a trial with manual feedback control to emulate a typical hyperthermia treatment workflow. In this trial, we maintained the MNP suspension at 43°C for approximately 10 min before allowing it to cool. Throughout these tests, the thermal camera was aimed at the side of the RF coil to monitor the temperature of the windings and capacitor bank.

### 2.6. Canine cadaver testing

To validate the combined MPI/MPH workflow, a canine cadaver was loaded with MNPs, imaged with CT and MPI, and heated with the new MPH system.

#### 2.6.1. Canine cadaver preparation and imaging

The dog was humanely euthanized in accordance with the Johns Hopkins University Animal Care and Use Committee (IACUC)-approved protocol and institutional guidelines. The skull was prepared by drilling burr holes in the left and right hemispheres and creating defects in the brain tissue similar to surgical brain tumor debulking to accommodate a MNP load. Approximately 0.5 ml of 50 nm MNPs at 80 mg/mL were added to each cavity and the access hole sealed with Barrigel^®^ (Teleflex, Wayne, PA). Cone-beam computed tomography (CBCT) (20s Head CBCT, Artis Zee, Siemens Healthineers, Forcheim, Germany) was performed after MNP implantation. The cadaver head was then frozen and transported to be imaged with MPI and tested in the new MPH system. A recently developed human head and neck MPI scanner, previously described in [27,39], was used to acquire tomographic MPI images after thawing. The cadaver head was then refrozen at −20°C.

#### 2.6.2. Canine cadaver nanoparticle heating test

The canine cadaver was partially thawed, and a small amount of hydrogel and tissue were removed to enable insertion of fiber optic temperature probes in each burr hole. We performed the testing before the head could completely thaw in order to preserve the structural integrity of the brain tissue, even though the latent heat effects from thawing would influence the initial temperature change. Our objective was to observe the relative rise in temperature between the two hemispheres. The probes were laterally stabilized using two small pieces of 1/4 in plastic tubing and two styrofoam blocks on top of the head (Figure 1(D)). The partially frozen head was wrapped in plastic and linen and positioned within the heating coil with the burr holes approximately along the axial center, based on tape measure and visual estimates. We monitored the temperature of each hemisphere during application of a *B*_*peak*_ =14mT field at 100.4 kHz for 20 min followed by no field for 15 min. While useful for experimental testing, we note that the size difference between the selected dog skull and a human head could result in variations in field homogeneity, eddy currents, and intracranial temperature distribution.

### 2.7. Computational modeling

To simulate a clinical treatment scenario using the head RF coil, we used a deidentified magnetic resonance imaging (MRI) human head dataset to perform a virtual treatment of a glioma patient. The scenario modeled the experimental work performed with the canine head cadaver, where a tumor with two different MNP injection sites was heated with an AMF. The human head dataset was processed and segmented to generate a 3D computational model. Details of model preparation, material properties, governing equations, and boundary conditions are provided in our previous work [44, 2025) and in the (Supplementary Information Tables S1–S5). We have verified and validated the solver for MPH heating previously [15,45]. The human head geometry consisted of the skull, cerebrospinal fluid (CSF), brain (white and gray matter averaged), CSF ventricles, and tumor. A ThermOmmaya reservoir was incorporated to represent MNP delivery [46]. The ThermOmmaya is a modified Ommaya reservoir with two ports to accommodate either MNP injection or temperature probes. Two 3.5 mm burr holes were introduced into the skull to mimic ThermOmmaya ports, and a biopsy cavity was created in the tumor to represent surgical coring.

Mathematically-derived ellipsoidal MNP distributions were assumed in each cavity, as shown in Figure S1, which mimicked the MNPs deposited in the canine cadaver. A conical extension was included at each ellipsoid to simulate MNP backflow during injection. MNP concentration was modeled similarly to a Gaussian profile, with the highest concentration at the ellipsoid center and decreasing toward the boundaries [45]. Heat was modeled as a volumetric source, which scaled with MNP concentration and had a peak value at the center of the ellipsoid of 5.0 × 10^6^ W/m^3^, corresponding to MNPs at a concentration of 50 mg/mL with a specific loss power (SLP) of 100 W/g Fe. See the Supplementary Information and Figure S1 for more information about the MNP distribution. Two multi-point fiber optic temperature probes were modeled, each inserted through a port and positioned along the axis of the corresponding ellipsoid toward the ThermOmmaya reservoir. The probes were modeled based on a multi-point fiber optic system (EVOLUTION, FISO Technologies, Ltd., Quebec, QC, Canada), with eight sensors spaced 3 mm apart. These virtual sensors were used to monitor temperature and thermal dose during treatment. Thermal dose was quantified as CEM43, and tumor coverage index (CI) was defined as the fraction of tumor volume reaching CEM43 ≥20 min. The goal of the treatment was to achieve CEM43 ≥20 min in at least 90% of the tumor volume, which is equivalent to CEM43T90 of 20 min, which has been shown to improve the effectiveness of thermal therapies [47]. In addition to the clinically feasible fiber optic probes, four virtual point probes were positioned 2 mm outside the tumor margin in the computational model. These additional probes were used to evaluate the thermal dose that would be delivered to the healthy tissue if treatment monitoring and control were based solely on the fiber optic probe measurements. The total simulation time was 50 min. We have previously verified and validated this model [45].

To obtain 95% confidence intervals, standard deviations for brain thermal properties and perfusion were obtained from the ITIS database [48] and assumed to be representative of tumor tissue. Only tumor thermal properties were varied, as these contribute the maximum uncertainty. The properties were adjusted by two standard deviations to capture the 95% confidence interval. Based on these bounds, two computational studies were performed to obtain lower and upper scenarios for temperature, thermal dose, and CI during treatment.

## 3. Results

### 3.1. Design verification

The device was verified against our design requirements, as summarized in Table 1, detailed below.

**Figure 2.**
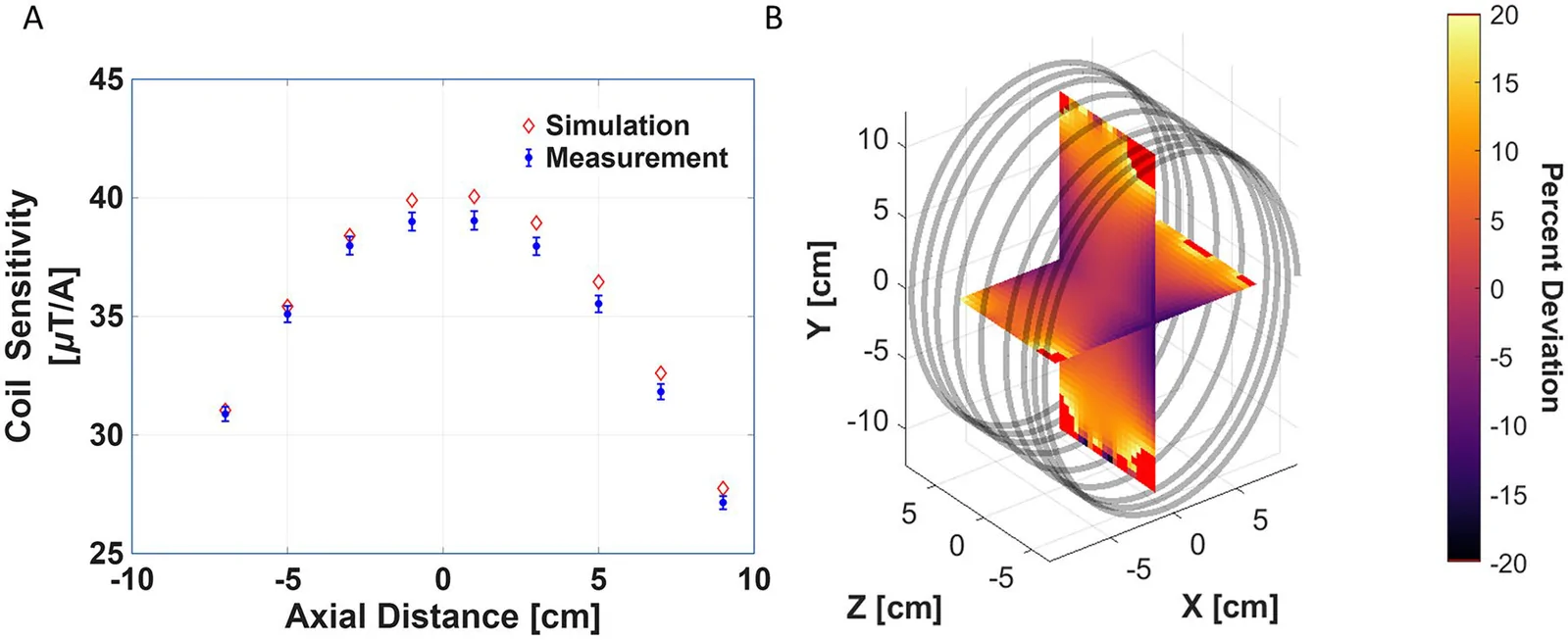
Simulated and measured field uniformity: A. Comparison between experimental field measurements inside the coil and the simulation results along the center axis (*X* = 0, *Y* = 0). Error bars for experimental measurement correspond to the precision of the current monitor (±1%); B. Simulated 2D field uniformity along the XZ and YZ planes. Values beyond ±20% are numerically clipped and shown as red.

We verified that the constructed coil fits an adult human head (Figure 1(C)) by test-fitting multiple adult subjects consisting primarily of members of the research team. The subjects noted that the coil was a tight fit and little clearance remained if access was needed to the patient’s head during treatment. Simulated and experimental field measurements confirmed that the coil met the field homogeneity requirements of ±10% along a 10 cm axial centerline. Figure 2(A) shows less than 3% error between the simulated and experimental magnetic field. The coil was wound as a spiral and was not radially symmetric, resulting in the maximum field being slightly offset from the coil isocenter (Figure 2(A)). Figure 2(B) displays the uniformity in the XZ and YZ planes. We attribute the mismatch between simulation and measured fields to accumulated manufacturing tolerances which arose because the device was hand-wound.

The measured coil inductance and resistance were consistent with simulation (Table 2), and the calculated voltage, current, and power dissipation at the coil were within the design envelope (Table 3). We note that the absolute difference between the simulated and measured resistance was 8.7 mΩ, which can reasonably be attributed to additional wire length needed for the connection leads, proximity losses due to eddy currents, or contact resistance from the impedance analyzer. Assuming a relative permeability of 1, a peak field of 14 mT at 100.4 kHz corresponds to a field x frequency product of 1.12×10^9^A /(m·s), which is below the Hergt-Dutz threshold of 5×10^9^ A /(m·s) [49] and coincides with other clinical scale systems described in Section 1.

### 3.2. Thermal testing in phantoms

We tested the MPH system using two phantoms: a concentration-gradient phantom and a tumor phantom. The concentration-gradient phantom was used to verify basic system operation and to provide qualitative guidance on the tracer concentrations required for clinical studies. The tumor phantom was designed to mimic expected performance during treatment.

#### 3.2.1. Nanoparticle heating: concentration gradient phantom

**Figure 3.**
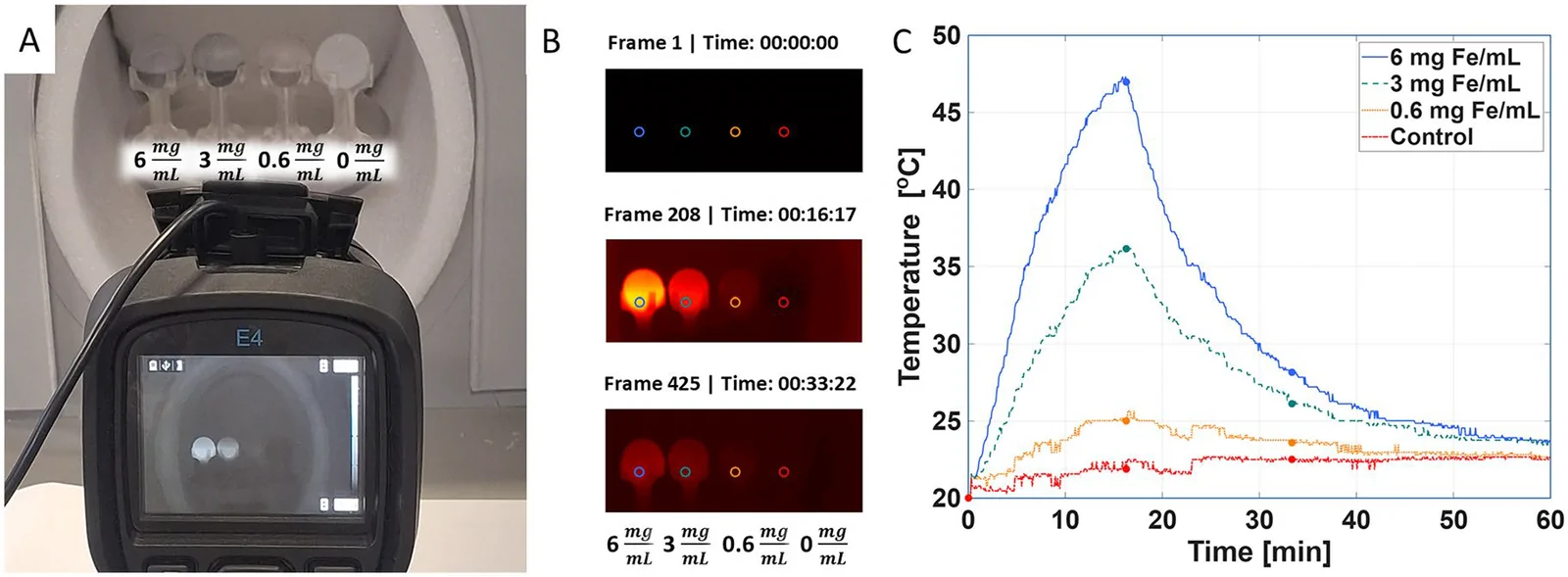
Measuring MNP heating within the coil at *B*_*peak*_ =11*mT* and 100.4 kHz using a thermal camera: A. Experimental setup using four different MNP samples of increasing concentration; B. Analysis of the thermal camera images with respect to time. Images from three different time points are shown; C. Temperature vs time plots for each vessel. The plotted values correspond to the measured temperatures in the circled regions in panel B. Reference markers (dots) indicate the frame corresponding to the images in panel B. The temperature plot shows the nominal pixel value of indicated region with an assumed uncertainty of ±2%.

We demonstrated MPH heating performance using a phantom containing multiple MNPs concentrations (Figure 3). The test measured the temperature rise using a thermal camera (Figure 3(A)). Representative frames from the thermal camera are shown in Figure 3(B), and the full temperature time course in Figure 3(C). The results of the test showed the MNPs heated in response to the applied field, where the change in temperature increased with MNP concentration. We observed a temperature rise of a 2.7°C in the 0 mg/mL (water) vessel, which we attributed to heat transfer from the nearby heated vessels and RF coil operation.

#### 3.2.2. Nanoparticle heating: tumor phantom

**Figure 4.**
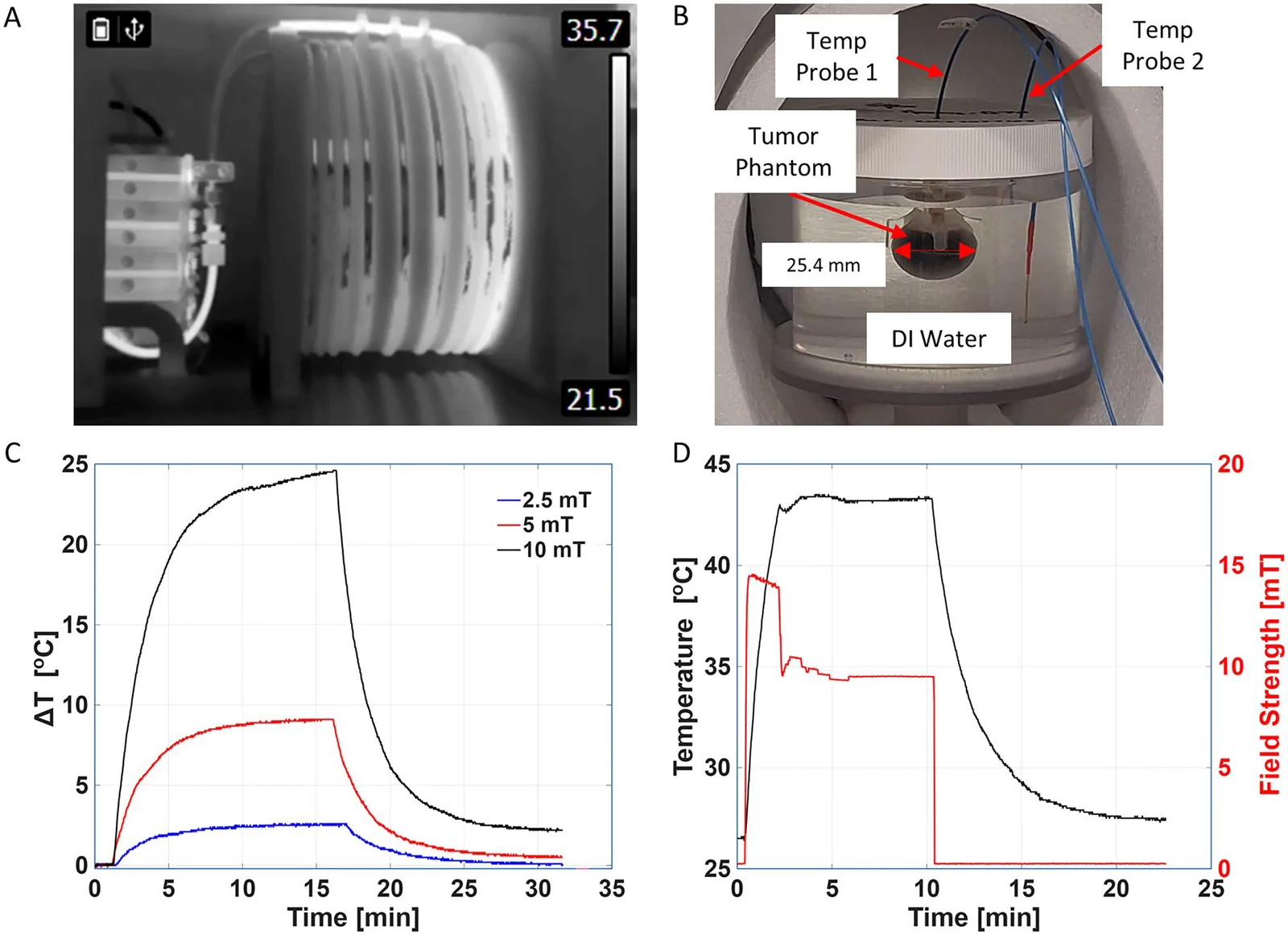
Heating of the tumor phantom: A. IR thermal image of the coil at peak operation during test found the water outlet temperature was 35.7°C; B. Photo of tumor phantom with temperature probe placement. A spherical vessel of MNPs was suspended from the lid of a container filled with room-temperature deionized water. One probe measured the change in temperature within the tumor phantom, while another measured any change in water temperature; C. Change in measured temperature at one intratumoral point (probe location) at increasing magnetic field strengths; D. Manual feedback control was able to rapidly reach and maintain the phantom temperature at 43°C. Thermal images shown in °C. Temperature and magnetic field strength plots have assumed uncertainties of ±0.2°C and ±1%, respectively.

Our measurements with the thermal camera indicated that water cooling was effective in maintaining the RF coil at reasonable temperatures during operation. During heating tests, we monitored the external coil temperature, and the highest temperature measured was ~35.7°C (Figure 4(A)). The tumor phantom loaded with MNPs and submerged in water (Figure 4(B)) heated as expected. As the magnetic field increased in amplitude (*B*_*peak*_) from 2.5 mT to 10.0 mT, the tumor phantom temperature change increased accordingly (Figure 4(C)).

We tested manual feedback control by adjusting the AMF amplitude to maintain the probe location at 43°C (Figure 4(D)). We began the test at *B*_*peak*_ =14.0 mT, which rapidly brought the tumor phantom temperature to 43°C. We then adjusted the AMF amplitude as needed to hold the temperature at 43°C for approximately 10 min before turning it off and monitoring the subsequent passive cooling of the phantom in the surrounding water bath. Figure S2 in the Supplementary Information shows the water temperatures measured.

### 3.3. MPI/MPH workflow validation using canine cadaver

**Figure 5.**
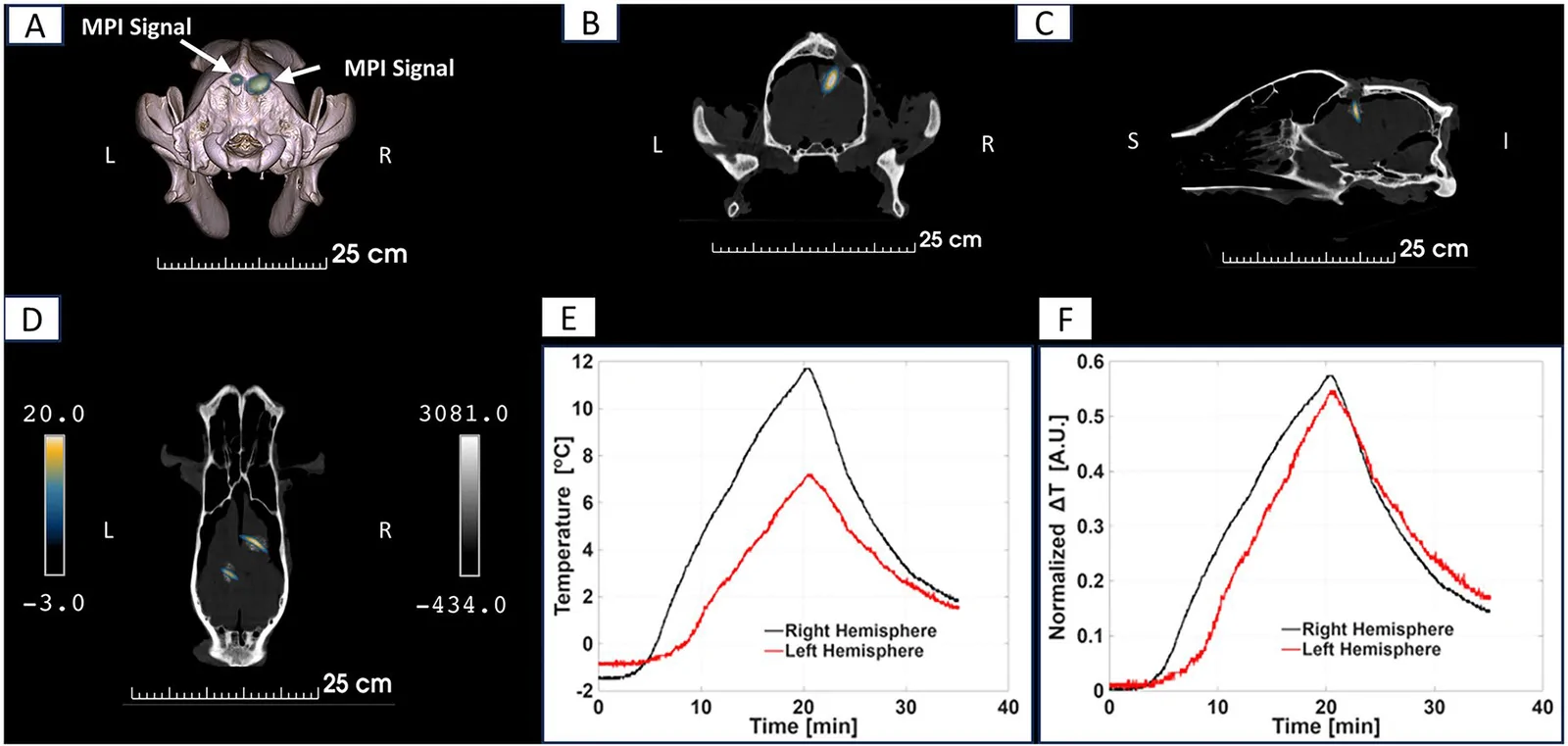
Validation of an image-guided MPI/MPH workflow in a canine cadaver. The MPI scan (blue/yellow colormap) is shown co-registered with a CBCT anatomical reference image (gray colormap) to provide a map of MNP location and quantity. A. Volume-rendered MPI/CBCT showing the right hemisphere (RH) injection with a larger signal than the left hemisphere (LH); B. Transverse (axial) slice centered on the RH injection; C. Median (sagittal) slice centered on the RH injection; D. Dorsal (coronal) slice showing both injections; E. Time course of heating with a *B*_*peak*_ =14.0*mT* field applied. The RH and LH injections heat at different rates, consistent with their MPI-detected iron content; F. Temperature rise above the minimum, normalized by peak MPI signal (RH = 23, LH = 15). Temperature plots have assumed uncertainties of ±0.2°C.

We validated an image-guided MPI/MPH workflow in a canine cadaver (Figure 5). The surgery and MNP administration were performed without complication. MNP location was confirmed by CBCT, which showed two hyperintense regions at the administration sites. MPI scanning was then performed, and the datasets were co-registered to yield the combined MPI/CBCT dataset shown in Figure 5(A–D). The MPI signal illustrated both MPI’s specificity and quantitation: the MPI signal co-localized with the CBCT hyperintensities, and the right-hemisphere (RH) signal amplitude was 1.53× higher than the left-hemisphere (LH) signal (RH/LH =1 53.).

Heating was performed after placing the fiber optic probes in the MNP-containing region in each hemisphere. The cadaver was heated at a field of *B*_*peak*_ =14 mT and 100.4 kHz (Figure 5(E)). Before heating, the cadaver was partially thawed so that the brain remained slightly below 0 C, consistent with partially frozen tissue. Upon AMF application, the temperature initially remained unchanged due to the latent heat of fusion as the tissue melted. After melting, the measured temperature increased in both hemispheres, with a larger temperature rise in the right hemisphere (≈12°C) than in the left (≈7°C; Figure 5(E)), consistent with the higher concentration observed in the MPI image.

To test MPI quantitation and its suitability for predicting heating, the temperature value for each hemisphere was normalized by dividing the temperature rise above its minimum value by the peak MPI signal at the corresponding injection site. After normalization, the resulting temperature curves (Figure 5(F)) were similar in both magnitude and slope. The normalized magnitudes differed by 4.7%, and the mean slopes between 700 s and 1200s (a period of approximately linear heating) differed by 8.9%.

### 3.4. Computational model

**Figure 6.**
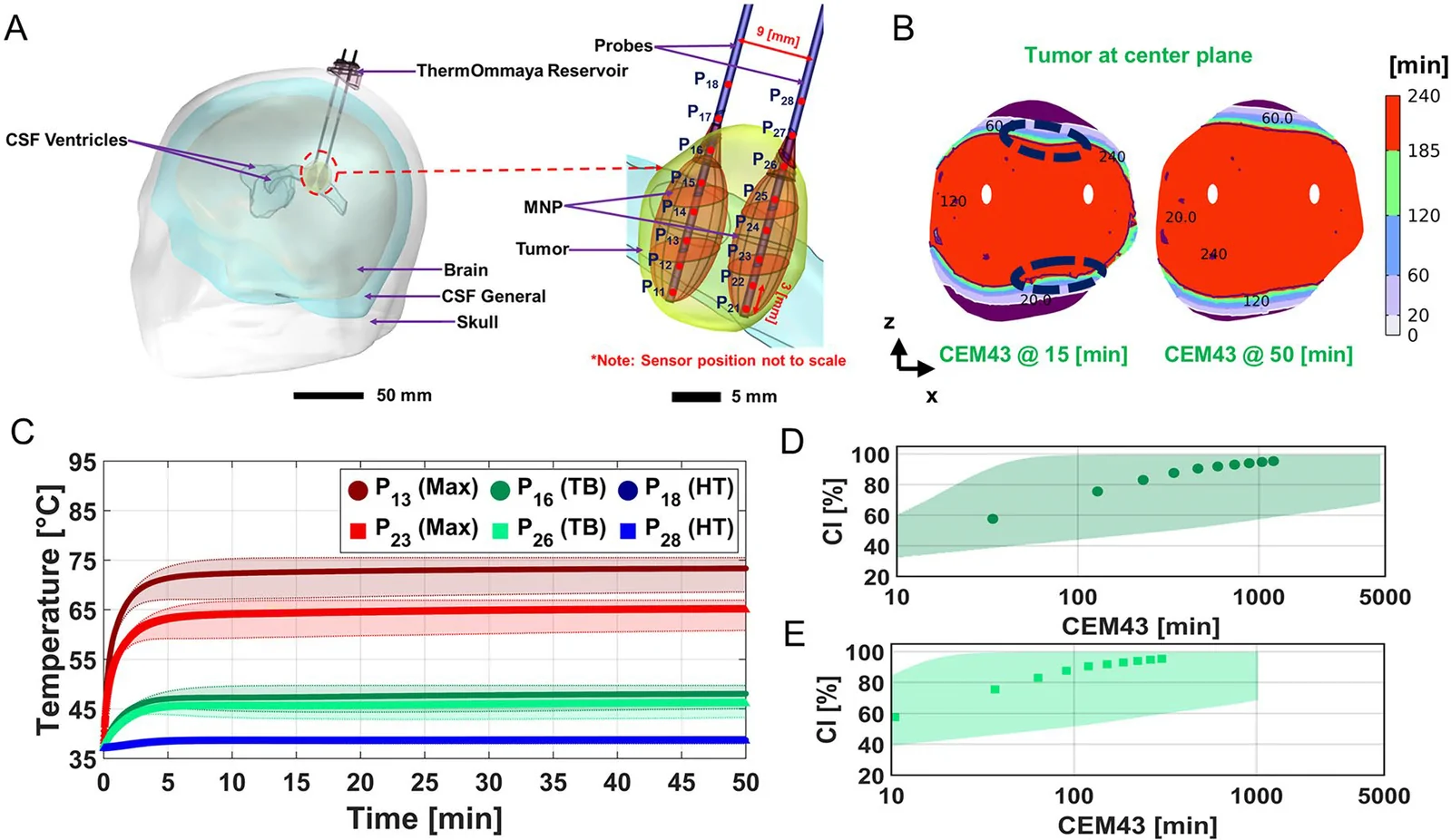
Computational simulation results for the deidentified human head model. A. 3D head geometry segmented from the patient dataset, showing ellipsoidal MNP distributions within the tumor region. The distributions were modeled assuming a surgical biopsy cavity and MNP delivery via ThermOmmaya reservoir ports. Multi-point fiber optic probes were included to simulate temperature and thermal dose measurements; B. Cross-sectional CEM43 maps at 15 and 50 min of simulated treatment. White regions in the middle are the location of the temperature probes; C. Temperature profiles at representative probe locations with 95% confidence regions shown: maximum treatment temperature (P_13_ and P_23_), tumor boundary (TB) temperature (P_16_ and P_26_), and healthy tissue (HT) sites (P_18_ and P_28_); D. CI vs CEM43 values with 95% confidence intervals at tumor boundary probe P_16_ and **E.** P_26_. Data points show mean values, shaded areas show 95% confidence intervals.

Simulation results of the glioma patient treatment (Figure 6(A)) show uniform heating within the tumor core, with thermal dose diffusing radially outward. After 15 min of treatment, an asymmetrical thermal dose distribution was observed between the two injection sites, attributed to the heterogeneous MNP distribution in the model (Figure 6(B)). The probes positioned within the regions of highest MNP concentration, P_13_ and P_23_, registered temperatures in the ablative temperature range (> 50°C), while probes along the tumor boundary P_16_ and P_26_, maintained temperatures within or slightly above the therapeutic hyperthermia range (41–45°C) (Figure 6(C)) [50]. Probes P_18_ and P_28_, located in surrounding healthy tissue, remained below 40°C throughout the simulation. Figure 6(C) also shows 95% confidence bounds propagated from material property uncertainty. Plots showing the temperature and thermal dose at all temperature probes modeled in the simulation are shown in Figure S3, where the maximum tumor temp can be seen in Figure S4. Calculation of thermal dose and CI from the temperature data (Figure 6(D,E)) revealed that, at the conclusion of the simulation (50 min), the CI reached 95% with the tumor boundary probes achieving high nominal CEM43 values of 1206 min (P_16_) and 303 min (P_26_). Due to the exponential relationship between thermal dose and intratumor temperature, the 95% confidence bounds shown in Figure 6(D) are large in relation to those in Figure 6(C). A hyperthermic rim (41–45°C) of ~4 mm was observed at the conclusion of the virtual treatment around the tumor boundary (Figure S5), where the four computational point probes randomly placed 2 mm outside the tumor margin achieved a CEM43 of 2.9 min, 4.5 min, 13 min, and 0.5 min.

## 4. Discussion

MPH therapy has technology-driven limitations, including uncertainty with the *in vivo* MNP distribution, reliance on invasive thermometry, and poor spatial control of the heating; these needs are reviewed in detail by [17]. Here, we address part of this gap by demonstrating key components of a clinical MPI/MPH workflow. In particular, we test at the clinical scale using MPI to directly measure MNP location and quantity. This new information enables the verification of MNP delivery and can serve as an input for patient-specific treatment planning. With further development and integration of MPI/MPH, combined instrumentation may one day solve these unmet needs and also support real-time thermometry and spatially confined energy deposition.

### 4.1. MPI and image-guided therapy

Our MPI results suggest an immediate role for MPI after MNP delivery to verify success by mapping particle location and amount. This capability is important because in prior preclinical work from our group, MNPs frequently redistributed after intratumoral injection due to leakage from the injection site and/or perfusion [20,51]. In the present study, the surgeon aimed to administer the same volume of MNPs to both treatment sites; however, MPI indicated a substantially higher signal in the right hemisphere than in the left, consistent with MNP loss from the left hemisphere during implantation or heating. Notably, this discrepancy was not apparent on CBCT, where both hyperintensities appear similar in brightness and volume. Beyond the post-delivery verification demonstrated here, MPI has also been demonstrated at video frame rates and could be used to monitor the MNP delivery itself and real-time changes in distribution during therapy [29,52].

MPI may also improve the accuracy of current temperature probes or reduce reliance on invasive thermometry altogether [53]. Our thermal simulations (Figure 6) indicated that accurate knowledge of the fiber optic probe location was critical in determining tumor thermal dose coverage and healthy tissue damage. Large tumors can require intense heating at their center to achieve adequate margins, as seen in Figure 6(C). In our canine cadaver head heating experiments (Figure 5), temperature probes were not placed under stereotactic guidance nor were they imaged after placement, so the measured temperatures likely did not capture the true maximum temperature within the cavity. Accurate co-registration of quantitative MPI with temperature probe location would enable accurate temperature measurement during therapy. In parallel, MPI-based thermometry methods are being developed that detect temperature-dependent changes from the MPI signal [54]. While not implemented here, such approaches could eventually complement or replace invasive probes in an integrated MPI/MPH system.

### 4.2. MPI for treatment planning

Our heating results support the usage of MPI as a key input for MPH treatment planning. At a basic level, quantitative MPI provides summary metrics (e.g. total MNP mass) that can be used to inform dosing or energy delivery before treatment. Our prior work explored this hypothesis, where we used MPI as an input to thermal simulations [22], as well as determined the MPI spatial resolution required for reliable treatment planning [45]. In this work, the MPI signal difference between the two intracranial injection sites correlated with the temperature change measured during heating (Figure 5(E,F)), which was expected given that thermal output scales linearly with MNP concentration and MPI signal is linear with MNP mass within a voxel [19]. After normalizing the temperature curves from each injection site to the corresponding MPI signal, the curves agreed closely, with less than 8.9% difference in temperature slope between the two brain hemispheres. While our study only analyzed the MPI scans and heating results of a single cadaver head (*N* = 1), the agreement between the MNP quantity and normalized heating rate is encouraging and reinforces the idea that MPI can be used to inform MPH treatment planning. Further study is needed with larger sample size to assess the impact of inter-subject variability.

Computational simulations assist with the treatment planning process by providing initial temperature predictions. For example, our simulated MPH treatment of a glioma (Figure 6(C)) predicted maximum intratumor temperatures that extended well into the ablative temperature range (Figure S4). Ablative temperatures during MPH are not without precedent in the literature, with both clinical [12] and preclinical [37] studies noting peak temperatures ≥ 50°C during heating. MPH requires a high intratumor MNP concentration in order to achieve the necessary heat generation, which consequently results in proportionally high intratumor temperatures. Some of our previous work suggests that a well-tuned temperature control system could help mitigate peak temperatures and avoid ablative tissue damage [15,55]. Additionally, simulations can help quantify the uncertainty associated with MPH therapy. As shown in our computational results (Figure 6(D)), the delivered dose at the tumor boundary has a wide uncertainty, due to large variations in assumed tissue properties. Given the exponential relationship between thermal dose and temperature, even small uncertainties in temperature can lead to large variations in CEM43 and, consequently, in the predicted CI. The largest contributor to model uncertainty is blood perfusion, where the standard deviation can vary as much as 20% relative to nominal values [48]. Prior work and the present results suggest that perfusion uncertainty alone can shift predicted temperatures by approximately 5–8°C [56]. Indeed, to improve the accuracy of thermal dosing, it can be beneficial to incorporate patient-specific perfusion maps into the model-based planning process. Further, models can even be run in real-time and used for advanced control strategies that improve treatment robustness and provide additional safeguards for keeping thermal dose within target limits [15].

### 4.3. Combined MPI/MPH for spatially confined heating

Integrating MPI/MPH can provide benefits beyond improved treatment planning and more accurate heating models by enabling spatially confined heating. MPI scanners produce images using a large gradient magnetic field to create a field-free region (FFR); MNPs inside the FFR interact with a transmit field and produce a signal, while MNPs outside this region are magnetically saturated and do not produce a signal. The same gradient field can be used for spatially confined heating, where MNPs within the FFR undergo hysteresis heating, and those outside the FFR are magnetically saturated and do not contribute to heating [23–26]. The FFR’s size and position can be adjusted, allowing for fine control of the heating area with the potential to be automated [57]. By integrating MPI and MPH in the same instrument, the gradient magnets can effectively “aim” the heating within a specific region. For example, considering our canine cadaver heating experiment, a spatially confined MPH system could deliver equal power to both brain hemispheres, target only one hemisphere while sparing the other, or focus heat on a specific tumor margin. In a more general sense, combining MPI/MPH in the same instrument can enable spatially-controlled MPH pulse sequences to selectively heat particles and improve the accuracy of the thermal dose delivered and treatment margins.

### 4.4. Need for clinically approved particles

At present, there are no MNPs approved for human use in MPH by the US FDA, and there is one particle/device combination approved by the EU (Nanotherm, Magforce NT, Berlin, Germany). Unfortunately, the iron oxide formulations used for imaging and iron replacement therapy, ferucarbotran and ferumoxytol, struggle to reach hyperthermic temperatures; this is seen in our previous work where we quantitatively compare the SLP between different MNPs [20]. Here, we utilized commercially available, preclinical-only MNPs optimized for MPH (synomag^®^, Micromod, Rostock, Germany), which have several benefits for combined therapy: possess a high SLP, available at concentrations of 1–10 mg/mL, and generate a strong MPI signal. As MPH technology developments continue, we anticipate further development and testing of MNP formulations better suited to combined MPI/MPH.

## 5. Conclusions and future design work

The designed MPI-compatible MPH sub-system was successfully built and tested through a variety of verification and validation experiments. We demonstrated that our human head coil generated a *B*_*peak*_ =10 mT field at 100 kHz within a 4 kW power envelope with a magnetic field homogeneity ±10% along a 10 cm axial centerline. Additionally, we showed the feasibility of a combined MPI/MPH workflow in one canine head cadaver by imaging and heating MNPs implanted in the brain. To explore if the coil design can be generalized to human treatment, we conducted thermal simulations using a human glioma model. With the coil design completed, our future work focuses on refining the coil design, testing spatially-confined heating at a clinical scale, and improving temperature feedback control. It is our hope that a combined MPI/MPH system can eventually play a role in bringing MPH treatment to further clinical testing.

## Supplementary Material

Supp 1

Supplemental data for this article can be accessed online at https://doi.org/10.1080/02656736.2026.2672017.

## Figures and Tables

**Table 1.** Design requirements, verification, and result.

Requirement	Verification	Result / Comment

Support MPH treatment of the adult brain	Tested fit on multiple adult subjects	While the coil successfully fit the tested adults (Figure 1(C)), it was tight and did not leave much room for probe placement
±10% Magnetic field homogeneity along 10 cm axial centerline	Measurement using magnetic field probe at multiple positions	The generated field matched the design and was 10% at ± 5 cm from isocenter (Figure 2(A))
Produce at least *B_peak_* = 10 mT at 100 kHz in a 4 kW power envelope	Magnetic field probe measured peak AMF	The RF coil produced *B_peak_* = 14 mT at 100.4 kHz, which exceeded our design goal (Figure 4(D))
Be suitable for future integration into the MPI imager	Test fit of coil into clinical MPI scanner; no use of magnetic materials	We successfully test fit the coil into the scanner (Figure 1(C)) and used only compatible materials

**Table 2.** Simulated and measured coil parameters at 100 kHz. The uncertainty in the measured values corresponds to the stated accuracy of the impedance analyzer when operated at 100 kHz.

	Simulated	Measured(±0.3%)

**Resistance [m**Ω**]**	35.0	43.7
**Inductance [** *μ* **H]**	15.5	15.6

**Table 3.** Operating-point estimates at 100 kHz assuming RF coil sensitivity of 40 μT/A. Operating voltage and copper loss are computed using the measured resistance and inductance values from Table 2.

Operating Point	*B_peak_* = 10 mT	*B_peak_* = 14 mT

**Estimated current [** *I*_peak_ **, A]**	250	350
**Operating voltage [** *V*_peak_ **, V]**	2451	3431
**Copper loss [*P*, W]**	1366	2677

## Data Availability

The data for this work are available here: https://doi.org/10.7281/T1LEZEKJ. Medical images used to create the clinical-scale thermal simulations can be made available upon request.
