# Supplementary material for: Verification and validation in canine cadaver of an MPI-integrable RF head coil for magnetic particle hyperthermia
Source: Int J Hyperthermia. Author manuscript; Available in PMC 2026 Sep 3. (PMC13541239; doi:10.1080/02656736.2026.2672017)
Supplement: Supplementary file 1 [file NIHMS2185027-supplement-Supp_1.zip › Supplement/thth-2025-0345-File008.pdf]

Verification and Validation in Canine Cadaver of an MPI-Integrable RF Head Coil for Magnetic Particle Hyperthermia

Hayden Carlton
Department of Radiation Oncology and Molecular Radiation Sciences
Johns Hopkins University School of Medicine, Baltimore, Maryland 21231, USA

Eli Mattingly
Magnetic Insight, Alameda, CA, 94502

Abdul Rahman Mohtasebzadeh
Magnetic Insight, Alameda, CA, 94502

Yash Sharad Lad
Department of Mechanical Engineering, School of Science, Engineering, and Technology, The Pennsylvania State University—Harrisburg, Middletown, PA, 17110

Shreeniket Pawar
Department of Mechanical Engineering, School of Science, Engineering, and Technology, The Pennsylvania State University—Harrisburg, Middletown, PA, 17110

Anilchandra Attaluri
Department of Mechanical Engineering, School of Science, Engineering, and Technology, The Pennsylvania State University—Harrisburg, Middletown, PA, 17110

Dara Kraitchman
Department of Radiology and Radiological Science, Johns Hopkins School of Medicine, Baltimore, MD, 21287
Department of Molecular and Comparative Pathobiology, Johns Hopkins School of Medicine, Baltimore, MD, 21287

Robert Ivkov
Department of Radiation Oncology and Molecular Radiation Sciences, Johns Hopkins School of Medicine, Baltimore, MD, 21230
Department of Oncology, Sydney Kimmel Comprehensive Cancer Center, School of Medicine, Johns Hopkins University, Baltimore, MD, 21205
Department of Mechanical Engineering, Whiting School of Engineering, Johns Hopkins University, Baltimore, MD, 21218
Department of Materials Science and Engineering, Whiting School of Engineering, Johns Hopkins University, Baltimore, MD, 21218

Patrick Goodwill
Magnetic Insight, Alameda, CA, 94502

Geometry

The biopsy cavity filled with MNPs was assumed to be two ellipsoidal injections cored through ThermoOmmaya with same size as the MNPs. Additionally, we assumed that the MNPs had a backflow with shape of frustum of a cone. The modelled dimensions for MNPs are shown in Table S1 and Table S2.

Table S1: Dimensions of modelled ellipsoidal MNPs.

Ellipsoid		1	2
Center [mm]	x	18.025	27.025
	y	67.000	67.000
	z	-2.000	-2.000
Semi-major axes [mm]	a	3.500	3.000
	b	3.500	3.000
	c	7.500	7.500
Rotation [°]		-30.000 @ x-axis	

Table S2: Dimensions of modelled backflow of MNPs.

Frustum of cone		1	2
Bottom position [mm]	x	18.000	27.000
	y	69.500	69.500
	z	4.000	4.000
Radius [mm]	Bottom	1.500	1.500
	Top	0.500	0.500
Height [mm]		4.000	4.000
Rotation [°]		-28.500@ x-axis	

Computational Modelling

Pennes' bioheat equation (Equations S1 and S2) was used to model heat transfer between the domains, provided as a bioheat transfer module in COMSOL [1]:

$$\begin{cases} \rho_i C_{p,i} \dfrac{\partial T}{\partial t} - \nabla(k_i \nabla T) = Q_{perf,i} + Q_{MNP} + Q_{met,i} & if\ i = MNP \\ \rho_i C_{p,i} \dfrac{\partial T}{\partial t} - \nabla(k_i \nabla T) = Q_{perf,i} + Q_{met,i} & else \end{cases} \quad \text{(S1)}$$

$$\mathrm{Q_{Perf,i}} = \mathrm{\rho_b C_{p,b} \omega_{b,i} (T_b - T)} \quad \text{(S2)}$$

where ρ is the density [kg·m^{-3}], C_p is the specific heat capacity [J(kg K)$^{-1}$], k is the thermal conductivity [W(m K)$^{-1}$], ρ_b is the blood density [kg·m^{-3}], $C_{p,b}$ is the specific heat capacity of blood [J(kg K)$^{-1}$], and ω_b is the microvascular blood perfusion [s^{-1}]. The subscript '*i*'

represents the domain under consideration i.e. tumor, MNP and healthy tissue. The tissue material properties used for the computational phantom and the source are listed in Table S3.

Table S3 Thermal properties for the human head anatomy.

**Part**		**Heat capacity, C_p $[J(kg \times K)^{-1}]$**	**Density, ρ $[kg \times m^{-3}]$**	**Thermal Conductivity, k $[W(m \times K)^{-1}]$**	**Dynamic Viscosity, μ $[Pa \times s]$**	**Velocity, u $[m \times s^{-1}]$**	**Ref.**
**Brain (grey & white matter)**		3630	1046	0.51	-	-	2-4
**Tumor**	Lower	3552	1044	0.53	-	-	2-4
	Nominal	3700	1056	0.57	-	-	5
	Higher	3845	1068	0.61	-	-	2-4
**CSF (general and ventricles)**	Nominal	4096	1007	0.62	7.84×10^{-4}	0.01	2,5
**Blood**	Nominal	3617	1050	0.52	3.65×10^{-4}	0.1	2-4

Modeling CSF:

To reduce the computational burden, the CSF general and ventricles were modeled as solid domains with convectively enhanced conductivity. Equation S3 was used to compute the Prandtl number, where μ is the dynamic viscosity [Pa·s]. The Grashof number was calculated using Equation S4, where *Ra* is the Rayleigh number [6,7]. These dimensionless numbers were used to calculate the Nusselt number *Nu*, given by Equation S5, assuming that the angle of inclination of the CSF ventricle from the vertical (θ) is 30º [6]. Equation S6 gives the convective heat transfer between the CSF and the tumor using the Nusselt number. The properties of the fluid and the numerical values of *Pr*, *Ra*, *Gr*, and *Nu* are listed in Table S4.

$$\mathrm{Pr} = \mathrm{C_p}\mu/\mathrm{k} \tag{S3}$$

$$\mathrm{Gr} = \mathrm{Ra}/\mathrm{Pr} \tag{S4}$$

$$\mathrm{Nu} = 0.67[\mathrm{Gr} * \mathrm{Pr} * \cos(\theta)]^{0.25} \tag{S5}$$

$$\mathrm{q} = -\mathrm{kNu}\nabla\mathrm{T} \tag{S6}$$

Table S4: Cerebrospinal Fluid properties and calculation of Nusselt Number [5,8].

$\mathbf{k}$ $[\mathbf{W}(\mathbf{m.K})^{-1}]$	$\mathbf{C_p}$ $[\mathbf{J}(\mathbf{kg.K})^{-1}]$	μ $[\mathbf{Pa.s}]$	$\mathbf{Pr}$	$\mathbf{Ra}$	$\mathbf{Gr}$	$\mathbf{Nu}$
0.62	4096	7.84×10^{-4}	5.179	4.79×10^{5}	9.27×10^{4}	17.01

<u>Modeling thermal dose metric:</u>

Hyperthermia treatments are based on the calculation of CEM43 to identify the thermal dose delivered during treatment (i.e., the duration of exposure). Hence, the CEM43 thermal dose for the tumor was modeled using Equation S7 [9].

$$\mathrm{CEM43} = \int_{t=0}^{t=\mathrm{final}} \mathrm{B}^{43°\mathrm{C}-\mathrm{T}(x,y,z,t)} \tag{S7}$$

In Equation S7, the constant *B* is an empirical value derived from the measurement of cells in culture. For human cells, its value is set to 0.5 when $T > 43$ [ºC], and 0.25 for $T \leq 43$ [ºC] [9-12].

<u>Modeling of thermal damage-dependent blood perfusion:</u>

To model the relationship between blood perfusion and thermal damage, we used the Arrhenius first order kinetics (Equation S8) [13,14]:

$$\mathrm{DS} = 1 - \exp\left(-\mathrm{A}\int_0^t e^{-\frac{E_a}{RT(t)}}\right)\mathrm{dt} \tag{S8}$$

where *DS* is the degree of vascular damage over time, *A* is the frequency factor [s^{-1}], E_a is the activation energy [$J\cdot mol^{-1}$] *and R* is the universal gas constant [$J(K\cdot mol)^{-1}$] and *T(t)* is the absolute tissue temperature [K] as a function of time. The degree of vascular

damage varied between 0 and 1, with 0 representing no vascular damage, and 1 representing complete vascular damage or damage-induced collapse. Based on this Arrhenius model, the thermal damage-dependent microvascular blood perfusion was modeled within the tumor, represented by $\omega_{\mathrm{b,tumor}}$ in Equation S9 [13-15].

$$\omega_{\mathrm{b,tumor}}(\mathrm{T}) = \begin{cases} \omega_{\mathrm{b,tumor}}(30\mathrm{DS}+1), (\mathrm{DS} \leq 0.02) \\ \omega_{\mathrm{b,tumor}}(-13\mathrm{DS}+1.86), (0.02 < \mathrm{DS} \leq 0.08) \\ \omega_{\mathrm{b,tumor}}(-0.79\mathrm{DS}+0.884), (0.08 < \mathrm{DS} \leq 0.97) \\ \omega_{\mathrm{b,tumor}}(-3.87\mathrm{DS}+3.87), (0.97 < \mathrm{DS} \leq 1.0) \end{cases} \quad \text{(S9)}$$

The values of the microvascular perfusion rates for different domains, along with the values of A and E_a, are given in Table S5.

Table S5: Blood perfusion rates and Arrhenius damage parameters.

Property		Value	Ref
Blood Temperature, T_b		310.15 [K]	16
Blood Perfusion in Skull, $\omega_{b,skull}$		$1.43 \times 10^{-4} [\mathrm{s}^{-1}]$	17
Blood Perfusion in Brain, $\omega_{b,brain}$		$8.50 \times 10^{-3}\ [\mathrm{s}^{-1}]$	17
Blood Perfusion in Tumor, $\omega_{b,tumor}$	Lower	$0\ [\mathrm{s}^{-1}]$	17,18
	Nominal	$1.72 \times 10^{-3}\ [\mathrm{s}^{-1}]$	17
	Upper	$5.185 \times 10^{-3}\ [\mathrm{s}^{-1}]$	17,18
Activation Energy, E_a		$2.38 \times 10^{5}\ [\mathrm{J.mol}^{-1}]$	15,19,20
Frequency Factor, A		$1.80 \times 10^{36}\ [\mathrm{s}^{-1}]$	15,19,20

Nanoparticle Distribution

Magnetic nanoparticle (MNP) distribution was modeled as two ellipsoidal domains representing injection from each ThermOmmaya reservoir port. The first ellipsoid measured 7.5 × 3.5 × 3.5 mm, and the second measured 7.5 × 3.0 × 3.0 mm, consistent with unequal deposition observed experimentally. To account for injection backflow, a conical extension was appended to each ellipsoid with a top radius of 0.5 mm, bottom

radius of 1.5 mm, and a height of 4 mm. MNP concentration was modeled similar to a Gaussian profile, with the highest concentration at each ellipsoid center and decreasing toward the boundaries. Once the ellipsoidal and conical domains were generated in the finite element software, they were exported as STL files and imported into MATLAB. A custom MATLAB function (provided below) was then used to generate randomized MNP distributions within each domain (Fig S1a and S1b). The resulting spatial distribution data were exported as spreadsheets and re-imported into the finite element software, where they were applied to the corresponding domains to simulate MNP heating.

The volumetric heat source within the ellipsoid scaled with concentration and assumed MNPs with an SLP of 100 [W/gFe]. This value was derived from our previous work [21], where we measured SLP of particles at 340 [kHz] and 10 [mT]. Assuming the established proportionality SLP $\propto H^2 f$, we calculated an effective SLP of approximately 100 [W/gFe] for Synomag 50 nm particles. The maximum MNP concentration was then iteratively varied in simulation until the final value of 50 [mgFe/mL] was chosen such that the resulting maximum treatment temperature remained below 80 [°C], noting that the maximum available stock concentration for these particles is 80 [mgFe/mL]. The product of SLP (100 [W/gFe]) and concentration (50 [mgFe/mL]) resulted in a peak volumetric thermal output of 5.0×10^6 W/m^3 at the center of the ellipsoid.

Initial and boundary conditions, and mesh and time step size:

Initially, all domains were assumed to be at baseline temperature of 37 [°C], corresponding to normal body temperature. A convective boundary condition was applied to the outer surface of the skull, with a convective heat transfer coefficient assumed at 10 [W/ (m^2.K)], and an ambient temperature of 20 [°C]. We used a physics-controlled tetrahedral mesh for which we have previously performed mesh sensitivity analysis for a heat source of 6.4×10^6 W/m^3. The number of tetrahedral elements for the study were 146589. The time step for the study was 1 s.

```
%%
clear; clc; close all;

%% ---------- USER SETTINGS ----------
stlPath        = 'stl path;
Npoints        = 30000;
edgeBand_mm     = 2.0;
gamma          = 1.0;
batchSize      = 1e5;
rng(1);

%% ---------- LOAD STL  ----------
S = stlread(stlPath); F=[]; V=[];
if isa(S,'triangulation')
   V = double(S.Points); F = double(S.ConnectivityList);
elseif istable(S) && all(ismember({'Points','ConnectivityList'},
S.Properties.VariableNames))
   V = double(S.Points{1}); F = double(S.ConnectivityList{1});
elseif isstruct(S)
   if all(isfield(S,{'Faces','Vertices'})), F = double(S.Faces); V = double(S.Vertices);
   elseif all(isfield(S,{'ConnectivityList','Points'})), F = double(S.ConnectivityList); V =
double(S.Points);
   elseif all(isfield(S,{'faces','vertices'})), F = double(S.faces); V = double(S.vertices);
   elseif isfield(S,'fv') && isstruct(S.fv) && all(isfield(S.fv,{'faces','vertices'}))
      F = double(S.fv.faces); V = double(S.fv.vertices);
   else, error('Unrecognized STL struct format from stlread.');
```

```
    end
else, error('stlread returned unsupported type: %s', class(S));
end
if size(V,2)~=3, error('Vertices not Nx3.'); end
if size(F,2)~=3, if size(F,1)==3 && size(F,2)>3, F=F.'; else, error('Faces not Mx3.'); end,
end
bad = any(isnan(F),2) | any(F<1,2) | any(F>size(V,1),2); F(bad,:)=[];
bad = F(:,1)==F(:,2) | F(:,2)==F(:,3) | F(:,1)==F(:,3); F(bad,:)=[];

%% ---------- BUILD SOLID ----------
shp = alphaShape(V, inf);

xmin=min(V(:,1)); xmax=max(V(:,1));
ymin=min(V(:,2)); ymax=max(V(:,2));
zmin=min(V(:,3)); zmax=max(V(:,3));

%% ---------- SAMPLE UNIFORMLY INSIDE THE SOLID UNTIL Npoints ----------
P = zeros(Npoints,3); k = 0;
while k < Npoints
    m = min(batchSize, Npoints - k);
    X = xmin + (xmax-xmin)*rand(m*3,1);
    Y = ymin + (ymax-ymin)*rand(m*3,1);
    Z = zmin + (zmax-zmin)*rand(m*3,1);
    inside = inShape(shp, X, Y, Z);
    Xi = X(inside); Yi = Y(inside); Zi = Z(inside);
    take = min(numel(Xi), Npoints - k);
    if take > 0
```

```
        P(k+(1:take),:) = [Xi(1:take), Yi(1:take), Zi(1:take)];
        k = k + take;
    end
end

%% ---------- DISTANCE TO SURFACE & CAPACITY  ----------
haveKNN = exist('knnsearch','file') == 2;
if haveKNN
    idx = knnsearch(V, P);
    dSurf = vecnorm(P - V(idx,:), 2, 2);
else
    nP = size(P,1); nV = size(V,1);
    chunk = max(1000, ceil(5e7 / max(nV,1)));
    dSurf = zeros(nP,1);
    for i = 1:chunk:nP
        j = min(i+chunk-1, nP);
        D = pdist2(P(i:j,:), V);
        dSurf(i:j) = min(D, [], 2);
    end
end

cap = dSurf / max(edgeBand_mm, eps);
cap = min(cap, 1).^gamma;

%% --- EXPORT ---
units = 'mm';
```

```
DATA = [P, cap];

fname1 = 'MNP_Distribution_Ellipsoid_1.txt';
fid = fopen(fname1, 'w');
fprintf(fid, '# columns: x_%s\ty_%s\tz_%s\tcap\n', units, units, units);
fprintf(fid, '%.6f\t%.6f\t%.6f\t%.6f\n', DATA.');
fclose(fid);

%% ---------- VISUALIZE ----------
figure('Color','w'); hold on; axis equal; view(3); grid on;

trisurf(triangulation(F,V), 'FaceColor',[0.8 0.8 0.85], ...
    'EdgeColor','none', 'FaceAlpha',0.22);
camlight; lighting gouraud;

s = scatter3(P(:,1), P(:,2), P(:,3), 6, cap, 'filled');
s.MarkerFaceAlpha = 0.9;

colormap(jet);
cb = colorbar; caxis([0 1]);
ylabel(cb, 'Intensity');

xlabel('X [mm]'); ylabel('Y [mm]'); zlabel('Z [mm]');
box on; axis vis3d;

fprintf('Filled %d points. Shell thickness = %.2f (units of STL). gamma = %.2f\n', ...
    Npoints, edgeBand_mm, gamma);
```

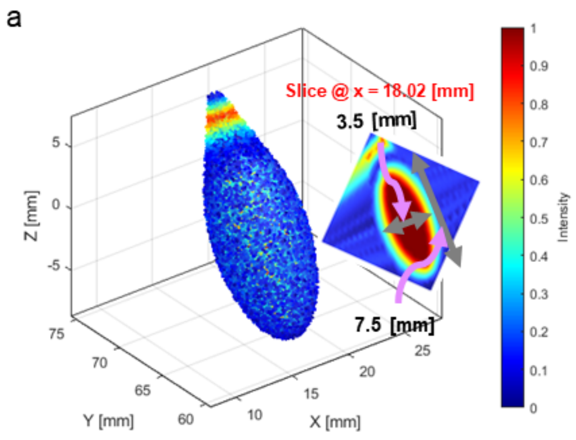

Figure S1: MNP distributions within the modeled conical and ellipsoidal domains. a. Distribution corresponding to the left ThermOmmaya port ellipsoid. B. Distribution corresponding to the right ThermOmmaya port ellipsoid.

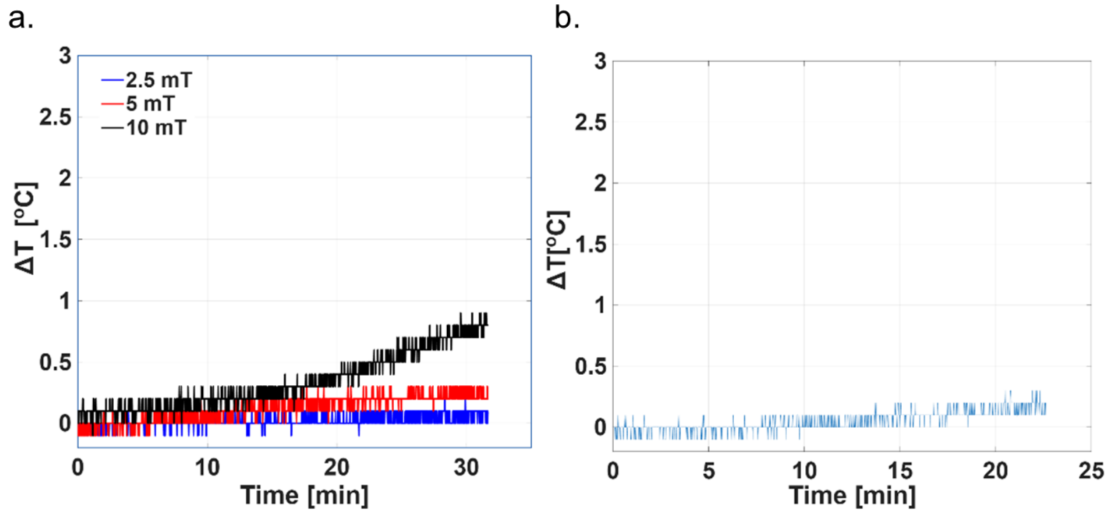

Figure S2: Water temperature during tumor phantom heating. a. Water temperature at different magnetic field settings (Figure 4c); b. Water temperature during modulated power test.

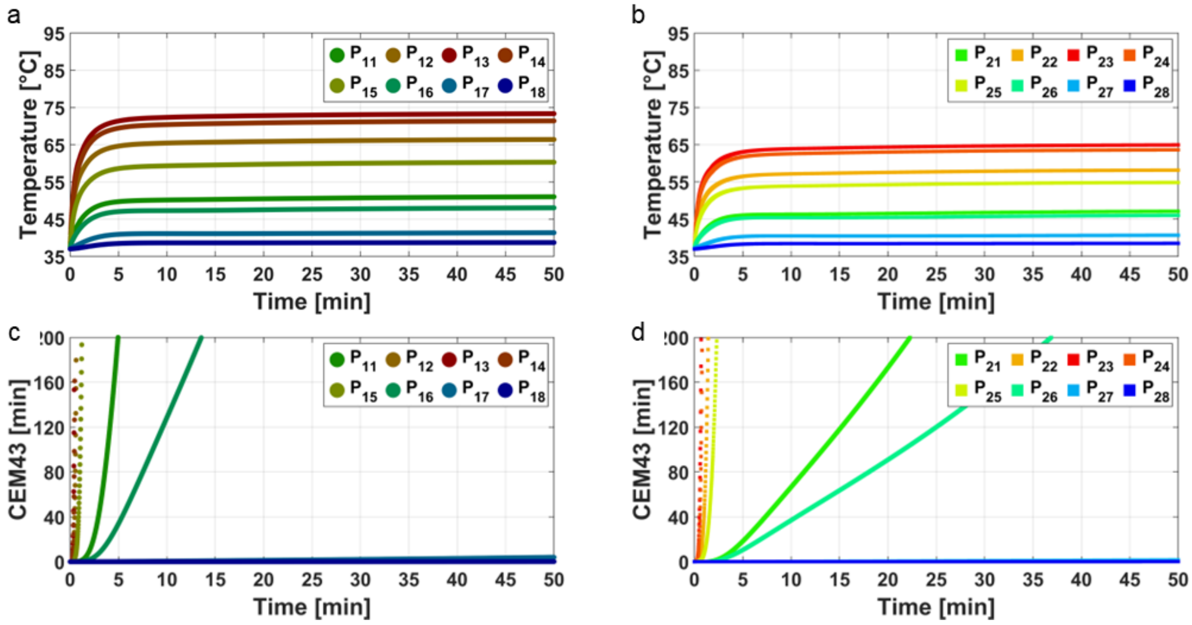

Figure S3: Temperature and CEM43 values calculated from the modeled fiber-optic probe sensors. a. Temperature measured by the left ThermOmmaya port probe sensors. b. Temperature measured by the right ThermOmmaya port probe sensors. c. CEM43 measured by the left ThermOmmaya port probe sensors. d. CEM43 measured by the right ThermOmmaya port probe sensors.

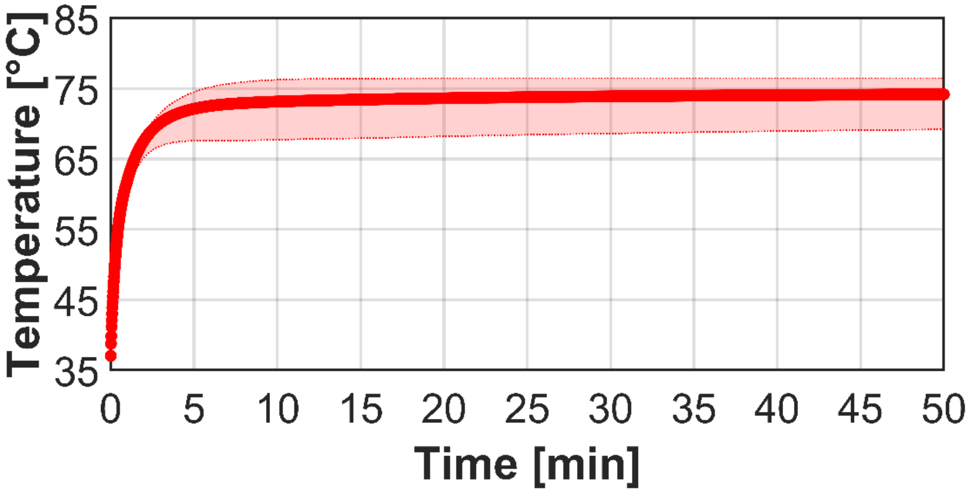

Figure S4: Maximum tumor temperature during simulated treatment.

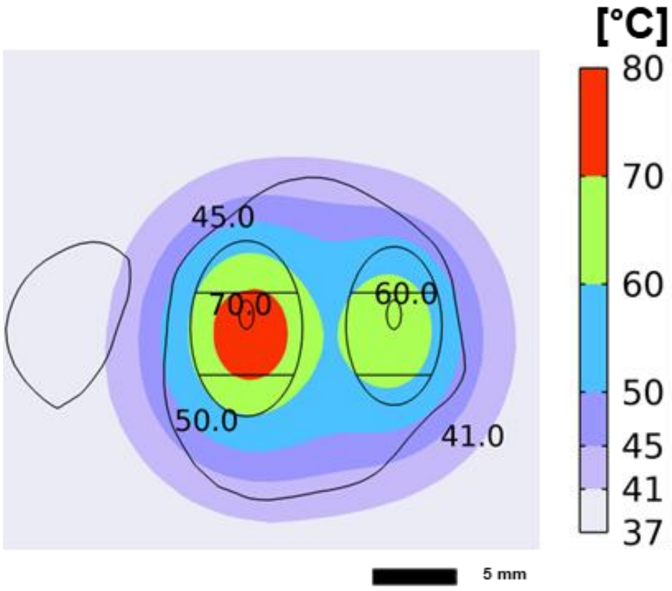

Figure S5: Hyperthermic rim around tumor boundary of approximately 4 mm observed after 50 minutes of computational treatment.

References

1. Pennes, H.H. Analysis of tissue and arterial blood temperatures in the resting human forearm. J. Appl. Physiol. 1948, 1, 93–122.
2. ITIS Foundation. Tissue Properties. Available online: https://itis.swiss/virtual-population/tissue-properties/database/database-summary/ (accessed on 12/27/2023).
3. ITIS Foundation. Heat capacity. Heat Capacity. Available online: https://itis.swiss/virtual-population/tissue-properties/database/heat-capacity/ (accessed on 12/27/2023).
4. ITIS foundation. Thermal conductivity. Available online: https://itis.swiss/virtual-population/tissue-properties/database/thermal-conductivity/ (accessed on 12/27/2023).
5. Schooneveldt, G.; Trefná, H.D.; Persson, M.; de Reijke, T.M.; Blomgren, K.; Kok, H.P.; Crezee, H. Hyperthermia Treatment Planning Including Convective Flow in Cerebrospinal Fluid for Brain Tumour Hyperthermia Treatment using a Novel Dedicated Paediatric Brain Applicator. Cancers 2019, 11, 1183.
6. Lim, C. K., & Chung, B. J. (2015). Natural convection experiments on the upward and downward faces of inclined plates using an electroplating system. Heat and Mass Transfer, 51, 713-722.
7. Nellis, G. F., & Klein, S. A. (2020). Introduction to Engineering Heat Transfer. Cambridge University Press.
8. Nellis, G., & Klein, S. (2008). Heat transfer. Cambridge university press. Pekar, L. (Ed.). (2020). Advanced Analytic and Control Techniques for Thermal Systems with Heat Exchangers. Academic press.
9. Sapareto, S. A., & Dewey, W. C. (1984). Thermal dose determination in cancer therapy. International Journal of Radiation Oncology* Biology* Physics, 10(6), 787-800.
10. Dewhirst, M. W., Viglianti, B. L., Lora-Michiels, M., Hanson, M., & Hoopes, P. J. (2003). Basic principles of thermal dosimetry and thermal thresholds for tissue damage from hyperthermia. International journal of hyperthermia, 19(3), 267-294.
11. Connor, W. G., Gerner, E. W., Miller, R. C., & Boone, M. L. (1977). Prospects for hyperthermia in human cancer therapy: Part II: Implications of biological and physical data for applications of hyperthermia to man. Radiology, 123(2), 497-503.
12. Dewey, W. C., Hopwood, L. E., Sapareto, S. A., & Gerweck, L. E. (1977). Cellular responses to combinations of hyperthermia and radiation. Radiology, 123(2), 463-474.
13. He, X., McGee, S., Coad, J. E., Schmidlin, F., Iaizzo, P. A., Swanlund, D. J., ... & Bischof, J. C. (2004). Investigation of the thermal and tissue injury behaviour in microwave thermal therapy using a porcine kidney model. International Journal of Hyperthermia, 20(6), 567-593.
14. Schutt, D. J., & Haemmerich, D. (2008). Effects of variation in perfusion rates and of perfusion models in computational models of radio frequency tumor ablation. Medical physics, 35(8), 3462-3470.
15. Kandala, S. K., Liapi, E., Whitcomb, L. L., Attaluri, A., & Ivkov, R. (2018). Temperature-controlled power modulation compensates for heterogeneous

nanoparticle distributions: a computational optimization analysis for magnetic hyperthermia. International Journal of Hyperthermia.
16. Nelson, D. A., & Nunneley, S. A. (1998). Brain temperature and limits on transcranial cooling in humans: quantitative modeling results. European journal of applied physiology and occupational physiology, 78, 353-359.
17. Gentilal, N., & Miranda, P. C. (2020). Heat transfer during TTFields treatment: Influence of the uncertainty of the electric and thermal parameters on the predicted temperature distribution. Computer Methods and Programs in Biomedicine, 196, 105706.
18. ITIS foundation. Heat Transfer Rate. Available online: https://itis.swiss/virtual-population/tissue-properties/database/heat-transfer-rate/ (accessed on 12/27/2023).
19. Rylander, M. N., Feng, Y., Zhang, Y., Bass, J., Jason Stafford, R., Volgin, A., ... & Diller, K. R. (2006). Optimizing heat shock protein expression induced by prostate cancer laser therapy through predictive computational models. Journal of biomedical optics, 11(4), 041113-041113.
20. Qin, Z., Balasubramanian, S. K., Wolkers, W. F., Pearce, J. A., & Bischof, J. C. (2014). Correlated parameter fit of Arrhenius model for thermal denaturation of proteins and cells. Annals of biomedical engineering, 42, 2392-2404.
21. Carlton, H., Salimi, M., Arepally, N., Bentolila, G., Sharma, A., Bibic, A., Newgren, M., Goodwill, P., Attaluri, A., Korangath, P., Bulte, J. W. M., and Ivkov, R. (2025). Ranking magnetic colloid performance for magnetic particle imaging and magnetic particle hyperthermia. Advanced Functional Materials, 35:2412321.
